# Improvement and application of vacuum-infiltration system in tomato

**DOI:** 10.1093/hr/uhae197

**Published:** 2024-07-26

**Authors:** Xinghao Yao, Ayat Taheri, Hang Liu, Yaojie Zhang, Ling Li, Jin Shao, Ke Wu, Qing Miao, Weizhi He, Xinyi Hu, Kexuan Tang

**Affiliations:** Joint International Research Laboratory of Metabolic and Developmental Sciences, Frontiers Science Center for Transformative Molecules, Plant Biotechnology Research Center, Fudan-SJTU-Nottingham Plant Biotechnology R&D Center, School of Agriculture and Biology, Shanghai Jiao Tong University, Shanghai 200240, China; Joint International Research Laboratory of Metabolic and Developmental Sciences, Frontiers Science Center for Transformative Molecules, Plant Biotechnology Research Center, Fudan-SJTU-Nottingham Plant Biotechnology R&D Center, School of Agriculture and Biology, Shanghai Jiao Tong University, Shanghai 200240, China; Joint International Research Laboratory of Metabolic and Developmental Sciences, Frontiers Science Center for Transformative Molecules, Plant Biotechnology Research Center, Fudan-SJTU-Nottingham Plant Biotechnology R&D Center, School of Agriculture and Biology, Shanghai Jiao Tong University, Shanghai 200240, China; Joint International Research Laboratory of Metabolic and Developmental Sciences, Frontiers Science Center for Transformative Molecules, Plant Biotechnology Research Center, Fudan-SJTU-Nottingham Plant Biotechnology R&D Center, School of Agriculture and Biology, Shanghai Jiao Tong University, Shanghai 200240, China; Joint International Research Laboratory of Metabolic and Developmental Sciences, Frontiers Science Center for Transformative Molecules, Plant Biotechnology Research Center, Fudan-SJTU-Nottingham Plant Biotechnology R&D Center, School of Agriculture and Biology, Shanghai Jiao Tong University, Shanghai 200240, China; Joint International Research Laboratory of Metabolic and Developmental Sciences, Frontiers Science Center for Transformative Molecules, Plant Biotechnology Research Center, Fudan-SJTU-Nottingham Plant Biotechnology R&D Center, School of Agriculture and Biology, Shanghai Jiao Tong University, Shanghai 200240, China; Joint International Research Laboratory of Metabolic and Developmental Sciences, Frontiers Science Center for Transformative Molecules, Plant Biotechnology Research Center, Fudan-SJTU-Nottingham Plant Biotechnology R&D Center, School of Agriculture and Biology, Shanghai Jiao Tong University, Shanghai 200240, China; Joint International Research Laboratory of Metabolic and Developmental Sciences, Frontiers Science Center for Transformative Molecules, Plant Biotechnology Research Center, Fudan-SJTU-Nottingham Plant Biotechnology R&D Center, School of Agriculture and Biology, Shanghai Jiao Tong University, Shanghai 200240, China; Joint International Research Laboratory of Metabolic and Developmental Sciences, Frontiers Science Center for Transformative Molecules, Plant Biotechnology Research Center, Fudan-SJTU-Nottingham Plant Biotechnology R&D Center, School of Agriculture and Biology, Shanghai Jiao Tong University, Shanghai 200240, China; Joint International Research Laboratory of Metabolic and Developmental Sciences, Frontiers Science Center for Transformative Molecules, Plant Biotechnology Research Center, Fudan-SJTU-Nottingham Plant Biotechnology R&D Center, School of Agriculture and Biology, Shanghai Jiao Tong University, Shanghai 200240, China; Joint International Research Laboratory of Metabolic and Developmental Sciences, Frontiers Science Center for Transformative Molecules, Plant Biotechnology Research Center, Fudan-SJTU-Nottingham Plant Biotechnology R&D Center, School of Agriculture and Biology, Shanghai Jiao Tong University, Shanghai 200240, China

## Abstract

The *Agrobacterium*-mediated transient expression system has been developed and applied to various plants as an alternative to stable transformation. However, its application in tomatoes is still limited due to low expression efficiency. In this study, we describe an improved vacuum-infiltration system that can be used in both tomato fruits and leaves. Notably, this study is the first report of vacuum infiltration in attached tomato fruits. The feasibility of the improved vacuum-infiltration system in Micro-Tom tomato was confirmed by various assays, including multiple fluorescent protein expression analysis, *β*-glucuronidase activity analysis, and *RUBY* reporter visualization. Subsequently, the improved vacuum-infiltration system was successfully applied to tomato biotechnology research. Herein, a trichome-specific promoter in tomato was identified that can drive the directional synthesis of specific plant natural products (PNPs). Additionally, based on the assessment results of the improved vacuum-infiltration system, we obtained a flavonoid-rich tomato variety through the stable transformation of *AmRosea* and *AmDelila*. In a significant practical application, we successfully synthesized the high-value scutellarin in tomato, which provides an alternative route for the production of PNPs from plants. In addition, the improved vacuum-infiltration system has been demonstrated to be suitable for commercial tomato varieties (‘Emerald’ and ‘Provence’) as well. The improved vacuum-infiltration system not only speeds up fundamental and applied research in tomato but also offers an additional powerful tool for advancing tomato synthetic biology research.

## Introduction

Tomato (*Solanum lycopersicum* L.) is an important economic crop and the second most cultivated vegetable in the world. It is rich in various high-value secondary metabolites, which make a substantial nutritional contribution to the human diet [1]. Over the past decades, the characterization of tomato gene function has been based mainly on establishing stable transgenic plants. With the development of plant biotechnology research, stable genetic transformation methods have been implemented in several tomato varieties, such as M82, Ailsa Craig, Money Maker, Micro-Tom, etc [2–4]. Nevertheless, a stable genetic transformation method is not suitable for all tomato varieties, especially for some commercially important ones (such as those serving as excellent chassis for plant biotechnology research) as they are recalcitrant to tissue culture and *Agrobacterium*-mediated transformation. In addition, the entire manipulation process remains laborious, time-consuming, and costly. These factors have restricted the development of tomato biotechnology research and tomato synthetic biology research. Therefore, developing fast, efficient, and high-throughput gene characterization strategies can help overcome these challenges.

The *Agrobacterium*-mediated transient expression system was initially applied to *Nicotiana benthamiana* leaves and has now been described in a variety of plant species such as *Populus davidiana* × *P. bolleana* [5], *Caragana intermedia* [6], *Theobroma cacao* [7], *Citrus macrophylla* [8], *Fragaria ananassa* [9], *Artemisia annua* [10], and *Persea americana* [11]. When compared to stable genetic transformation, the vacuum-infiltration system does not involve tedious tissue culture process and enables the results to be presented within 3–5 days, which particularly is suitable for plants with difficult regeneration establishment systems and those with long growth cycles. The vacuum-infiltration system has been widely used for promoter analysis [12], plant-pathogen interaction [13], transcription factor (TF) activity analysis [14], protein–protein interaction [15], protein subcellular localization [16], study of metabolic pathways [17], secondary metabolites production [18], and pharmaceutical proteins production [19]. Also, with the development of plant synthetic biology, vacuum-infiltration system is widely used for screening elements and testing biosynthetic pathways in plants [20]. To date, syringe infiltration is one of the most commonly used methods for the vacuum-infiltration system. However, the transformation efficiency of syringe infiltration depends on plant characteristics, so that thick stratum corneum and wax coat, low stomatal density or small aperture of stomatal pores, complex vein network arrangement, overall fragility of plant tissues, and small cellular gaps within the plant tissue often lead to low efficiency of syringe infiltration [2, 6, 21]. To address these issues, vacuum infiltration is gradually being developed for these species. For instance, an *Agrobacterium*-mediated vacuum infiltration method was developed in *Diospyros kaki* and used to investigate gene function [21]. In addition, there have been some reports on production of terpenes in *N*. *benthamiana* [22], as well as the manufacturing of 10 million doses of pandemic influenza vaccine per month in *N*. *benthamiana* based on vacuum infiltration by Medicago’s facility in North Carolina, USA [23].

**Figure 1:**
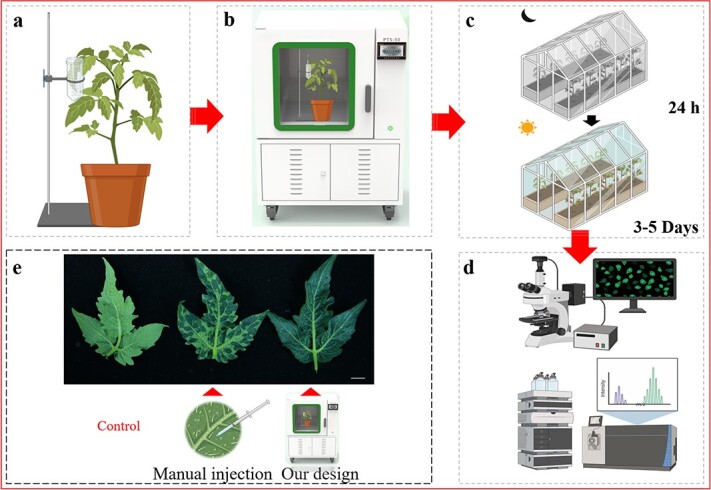
Schematic diagram of the experimental procedures for the improved vacuum-infiltration system in tomato leaves. **a** The tomato leaves were immersed in a centrifuge tube containing *Agrobacterium tumefaciens.*  **b** Plant vacuum transformation device (Model: PTS-50). **c** Greenhouse. Top, dark room; bottom, standard 16 h light/8 h dark room. **d** Multiple experimental analyses. **e** Phenotypic differences between syringe-infiltrated and vacuum-infiltrated tomato leaves (scale bars = 1 cm). Left, control group; middle, manual injection; and right, vacuum transformation.

Despite a series of advances in transient gene expression methods in plants, few have been reported in tomatoes. Due to their topology (tissue compactness, internal pressure, innervation pattern, etc.), syringe infiltration only allows protein expression in certain sections of the fruits and leaves [24–27]. To overcome the effect of tomato tissue structure on transient expression efficiency, the researchers also attempted to establish vacuum infiltration methods in tomatoes. For tomato leaves, although two vacuum infiltration methods have been reported sequentially, they are only suitable for sprouts or 3-week-old seedlings [28, 29]. For tomato fruits or other tissues, there are also a few reports on the establishment of vacuum infiltration methods, but they are limited to tomato detached tissues [30, 31]. However, because the detached tissues are unable to obtain the continuous supply of nutrients from plants, they are susceptible to rot after infiltration [30], which significantly affects the accuracy of the results. Up to now, there is no report on vacuum infiltration methods applicable to attached tomato fruits.

To further advance both fundamental and applied research in tomato, we established an efficient vacuum infiltration method for several attached tomato tissues. In addition to exploring the optimal expression period of exogenous genes in tomato leaves and fruits, we also carried out several biotechnology explorations, including element mining, element screening, and efficient synthesis of high-value plant natural products (PNPs).

## Results

### Improvement of vacuum-infiltration system in tomato

In general, most of the tomato transient transformation studies have been conducted on Micro-Tom (Fig. 1a), which is a conventional model plant. However, the structural traits of tomato leaves lead to inferior syringe infiltration (Fig. 1e). In order to enhance the efficiency of infiltration for transient assays, we developed a plant vacuum transformation device (Fig. 1b), which enables rapid and efficient infiltration of living leaves without damaging them. As shown in Fig. 1e, this device allows the leaf to be infiltrated with *Agrobacterium* suspension in a short time compared to conventional syringe infiltration. The improved vacuum-infiltration system is convenient to perform, and its process consists of four main steps (fixation, infiltration, dark/light incubation, and analysis; Fig. 1a–d).

### The improved vacuum-infiltration system is applicable to tomato leaves

**Figure 2:**
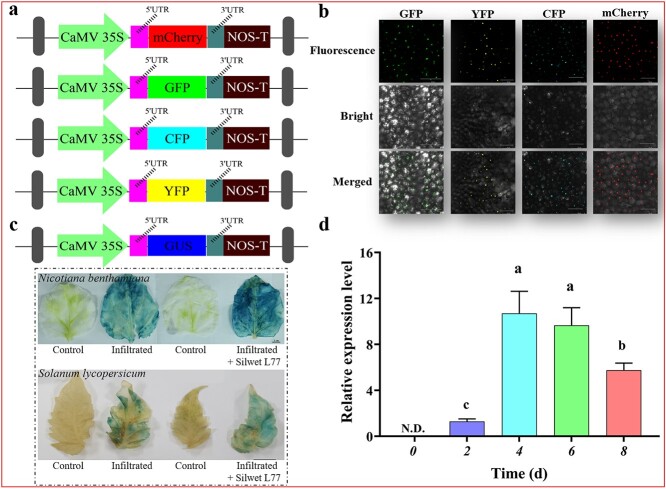
Transient expression analysis of GUS and multiple fluorescent proteins. **a** Schematic representation of different constructs used for the multiple fluorescent protein assay. **b** Confocal images of EGFP/YFP/CFP/mCherry expression in tomato leaves. Scale bars, 50 μm. **c** A schematic representation of the GUS construct and representative histochemical staining images showing GUS activity in *Nicotiana benthamiana* and tomato leaves. Top, *N. benthamiana* leaves; bottom, tomato leaves. ‘Infiltrated + Silwet L-77’ indicates leaves were infiltrated with *Agrobacterium* solution containing 0.02% Silwet L-77. Scale bars, 1 cm. **d** qRT-PCR analysis of *GUS* gene expression in tomato leaves. *SlTIP41* was used as the internal reference gene. Values are mean ± SD (*n* = 3). The different letters indicate statistically significant differences between groups (Tukey’s honest significant difference test, *P* < 0.05). N.D. = not detected.

To evaluate whether the improved vacuum-infiltration system could be well established, we first examined the expression level of four different reporter genes (enhanced green fluorescent protein, EGFP; yellow fluorescent protein, YFP; cyan fluorescent protein, CYP; and red monomeric fluorescent protein, mCherry) in tomato leaves. The vector pAGM4723-EGFP/YFP/CFP/mCherry was constructed with the cauliflower mosaic virus 35S promoter +5′-UTR (CaMV35S-P + 5 U) and the 3′-UTR + nopaline synthase terminator (3 U + NOS-T; Fig. 2a). As shown in Fig. 2b, the EGFP fluorescence signal intensity was clearly visible in tomato leaves 4 days post-infiltration (4 dpi), and the corresponding fluorescence was also observed in the other three fluorescent proteins. The confocal images indicated that fluorescent proteins were able to accumulate successfully in tomato leaves. To further support this conclusion, the *β*-glucuronidase (GUS) reporter gene was also evaluated as another research subject. Similarly, we constructed the pAGM4723-GUS vector (Fig. 2c), which was also transiently transformed into tomato leaves via the improved vacuum-infiltration system. Histochemical staining showed that large amounts of GUS proteins were detected in tomato leaves, which was consistent with the results of the positive control group (*N*. *benthamiana*; Fig. 2c). As expected, no GUS staining was detected in the control group (*Agrobacterium* solution with pAGM4723 only; Fig. 2c). It is worth noting that the presence of Silwet L-77 significantly improved the efficiency of transient expression (Fig. 2c). Taken together, both the fluorescent protein assay and the GUS staining assay demonstrated the stability and reliability of the improved vacuum-infiltration system and its applicability in tomato leaves. Moreover, with *GUS* as a reporter gene, we also explored the changes in the expression pattern of *GUS* gene in tomato leaves. As illustrated in Fig. 2d, *GUS* gene expression was detectable at 2 d, increased to a peak level at 4 d, and then decreased by 6 d.

### Application and testing of the improved vacuum-infiltration system in attached tomato fruits

**Figure 3:**
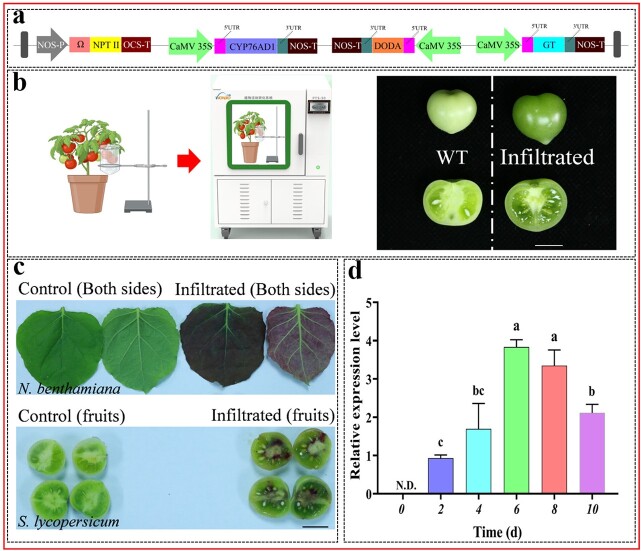
Construction of betalain biosynthesis pathway in attached tomato fruit. **a** Schematic representation of the structure of the three key enzymes necessary for betalain biosynthesis. _L_-DOPA, _L_-3,4-dihydroxyphenylalanine; DODA, _L_-DOPA 4,5-dioxygenase; GT, glucosyl transferase. **b** Application of efficient transient expression system in tomato fruits. Scale bars, 1 cm. **c** Synthesis of betalain in *Nicotiana benthamiana* leaves (top) and tomato fruits (bottom). Scale bars, 1 cm. **d** qRT-PCR analysis of *DODA* gene expression in tomato fruits. *SlTIP41* was used as a normalized control gene. Data are presented as the mean ± standard error of three independent biological replicates. The different letters indicate statistically significant differences between groups (Tukey’s honest significant difference test, *P* < 0.05). N.D. = not detected.

Tomato fruit is a model for fleshy fruit development, and research on the molecular basis of its growth and development is of increasing interest to botanists [32]. To establish the fruit transient expression system to assist tomato research, we also attempted to apply the improved vacuum-infiltration system to attached tomato fruits. As depicted in Fig. 3b, tomato fruits were kept in the vacuum transformation device after being immersed in *Agrobacterium* solution, which contained the target genes. The entire fruit would be filled with *Agrobacterium* following the transformation.

Betalain has a bright maroon color and can be used as a visual reporter to determine whether the protein is successfully expressed in the host cell. The biosynthesis of betalain has been extensively studied, and three enzymatic reactions are required to convert tyrosine to betalain [33]. The artificial open reading frame (ORF) of 2A-linked three betalain biosynthesis genes is named *RUBY* [33]*.* To further test whether the improved vacuum-infiltration system could enable the production of functional proteins, the betalain biosynthetic pathway was reconstituted in attached tomato fruit. In this study, in order to maximize the function of each gene, they were constructed separately in an expression cassette consisting of the CaMV35S-P + 5 U and the 3 U + NOS-T (Fig. 3a). Simultaneously, this work was repeated on *N. benthamiana* leaves as the positive control. As expected, tomato fruits showed the same maroon color as *N. benthamiana* leaves (Fig. 3c), indicating that betalain was successfully produced; the production of betalain in this study proved that all three key enzymes function simultaneously in the attached tomato fruit, demonstrating that the improved vacuum-infiltration system is also applicable to tomato fruits. In addition, qRT-PCR assays for the *DODA* gene were performed 0–10 days after infiltration to determine the time period required for optimal gene expression in tomato fruits. Results showed that the *DODA* gene was continuously upregulated from 2 d to 6 d, and the maximum expressed level appeared at 6 d. Surprisingly, *DODA* gene expression can continue for more than one week after *Agrobacterium* infiltration (Fig. 3d).

### *AaLTP* promoter is a candidate element for synthetic biology in tomato trichomes

**Figure 4:**
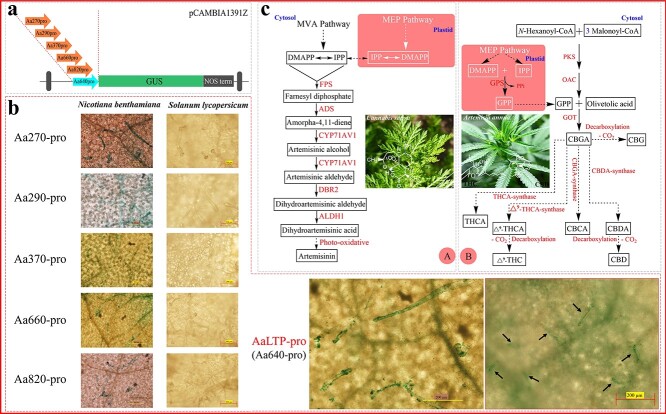
Screening of trichome-specific promoters in tomato. **a** Schematic of the T-DNA of the pCAMBIA1391Z vector used for GUS staining assay. **b** Histochemical localization of GUS expression (driven by different trichome-specific promoters of *Artemisia annua*) in *Nicotiana benthamiana* and tomato. Bars = 200 μm. **c** Biosynthetic pathways of artemisinin (A) and cannabinoids (B). ADS, amorpha-4,11-diene synthase; ALDH1, aldehyde dehydrogenase; CBCA, cannabichromenic acid; CBD, cannabidiol; CBDA, cannabidiolic acid; CBG, cannabigerol; CBGA, cannabigerolic acid; CYP71AV1, cytochrome P450 monooxygenase; DBR2, artemisinic aldehyde D11 [13] reductase; DMAPP, dimethylallyl pyrophosphate; FPS, farnesyl diphosphate synthase; GPP, geranyl pyrophosphate; IPP, isopentenyl pyrophosphate; MEP pathway, methylerythritol 4-phosphate pathway; MVA pathway, mevalonate pathway; THC, tetrahydrocannabinol; THCA, tetrahydrocannabinolic acid.

After the successful establishment of the improved vacuum-infiltration system in tomatoes, tissue-specific promoters were explored to further enrich the tomato synthetic biology element pool. Based on our previous research in the field of basic research and secondary metabolism of *A. annua*, six genes that were highly expressed in the *A. annua* trichomes were screened by analysing the transcriptomic data. As shown in Fig. 4a, the promoter region of each gene was constructed into pCAMBIA1391Z vector for GUS enzyme activity analysis, respectively. In the leaves of *Aa270pro* and *Aa640pro*-expressing *N. benthamiana*, intense GUS staining was observed in the trichomes. In the cases of *Aa290pro*, *Aa370pro*, *Aa660pro*, and *Aa820pro*, only slight GUS staining in the trichomes was observed. In contrast, GUS staining of trichomes was only observed in *Aa640pro*-expressing tomato leaves (Fig. 4b). Moreover, according to the BLAST (https://blast.ncbi.nlm.nih.gov/Blast.cgi) results, we found that Aa640 is the previously reported lipid transfer protein, a protein with the ability to transfer lipids between membranes [34]. The results indicate that *AaLTP-pro* was able to drive the expression of exogenous genes in tomato trichomes and could be a candidate element for tomato synthetic biology studies.

### The combination of *AmRosea* and *AmDelila* is a potent element for the creation of tomatoes with high flavonoid content

**Figure 5:**
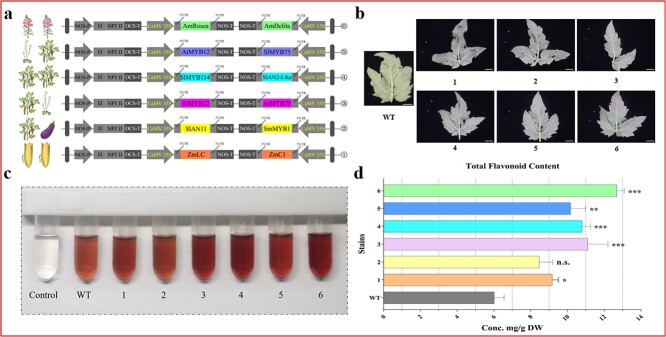
Different combinations of TFs promote the accumulation of flavonoids in tomato leaves. **a** Schematic representation of different TF combinations. **b** Phenotypes of flavonoid accumulation in leaves from plants of WT and vacuum-infiltrated. **c** Determination of TFC by aluminum chloride colorimetric assay. **d** Quantitative analysis of TFC of leaves from WT and vacuum-infiltrated tomatoes. Data are presented as the mean ± standard error of three independent biological replicates. Asterisks indicate statistically significant differences (^*^P < 0.05, ^***^P < 0.001; *t*-test); n.s. = not significant. The symbols in all figures correspond to the serial numbers in Fig. 5a. Scale bars, 1 cm.

Tomato is an important vegetable worldwide, and their levels of flavonoids are considered suboptimal [35]. To increase the flavonoid content in tomatoes, we suggested identifying TFs that can be applied to tomatoes using the improved vacuum-infiltration system. According to the existing studies on the regulation of TFs in flavonoid synthesis, 12 genes were selected. The origin of the different TFs and the six combinations are shown in Fig. 5a. To characterize the extent of their contribution to flavonoid accumulation, each of the six combinations was transiently expressed in tomato leaves using the improved vacuum-infiltration system. As shown in Fig. 5b, leaf 1^#^ to leaf 6^#^ exhibited different phenotypes, with leaf 3^#^ (*SlMYB12* + *AtMYB75*), leaf 5^#^ (*AtMYB12* + *SlMYB75*), and leaf 6^#^ (*AmRosea* + *AmDelila*) exhibiting more intense red coloration (Fig. 5b). Consistent with the observations from the phenotypes, the NaNO_2_-Al (NO_3_)_3_ colorimetric assay indicated that leaf 3^#^, leaf 5^#^, and leaf 6^#^ contained more flavonoids (Fig. 5c). Flavonoid quantitative results showed that only leaf 2^#^ leaves had no significant difference in total flavonoid content (TFC) compared with WT, and the TFC of the other five combinations (leaf 1^#^, leaf 3^#^, leaf 4^#^, leaf 5^#^, and leaf 6^#^) was significantly increased by 1.52-, 1.84-, 1.79-, 1.69-, and 2.11-fold, respectively. Notably, the TFC of leaf 6^#^ was the highest at 12.68 mg/g (Fig. 5d). These results suggest that the combination of *AmRosea* and *AmDelila* is a candidate element that can be applied for the development of high-flavonoid tomato varieties.

**Figure 6:**
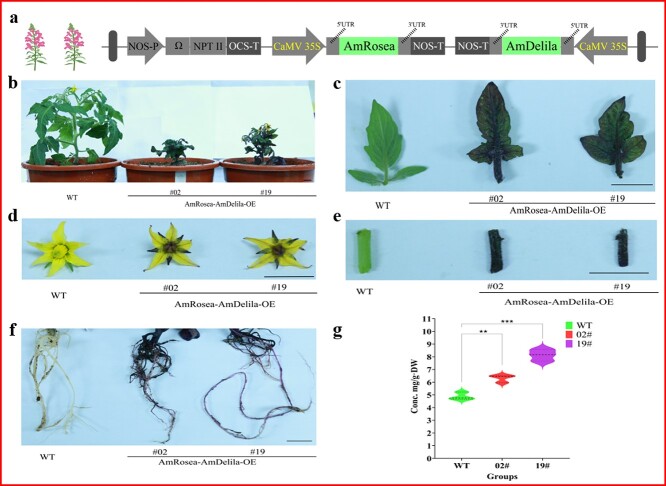
Large amounts of flavonoids accumulated in the *AmRosea*-*AmDelila*-OE lines. **a** Schematic representation of the gene cassette used to overexpress *AmRosea*-*AmDelila* in tomato. **b**–**f** Representative photographs of different tissues of the WT and *AmRosea*-*AmDelila*-OE lines. **b** plant. **c** leaf. **d** flower. **e** stem. **f** root. **g** Determination of TFC in leaves of WT and *AmRosea*-*AmDelila*-OE lines. Data are presented as the mean ± standard error of three independent biological replicates. Asterisks indicate statistically significant differences (^*^P < 0.05, ^***^P < 0.001; *t*-test). Scale bars, 1 cm.

To further characterize the role of *AmRosea* and *AmDelila*, the combination was stably transformed into tomatoes (Fig. 6a). As shown in Fig. 6c–f, the *AmRosea*-*AmDelila*-over expression (*AmRosea*-*AmDelila*-OE) lines accumulated large amounts of anthocyanins in both vegetative and reproductive organs, including flowers, stems, leaves, and roots, implying that massive amounts of flavonoids were present as substrates in the overexpression lines. Determination of flavonoid accumulation showed that the TFC in *AmRosea*-*AmDelila*-OE-#19 leaves was 8.13 mg/g (dry weight), which was 1.67 times higher than that in WT leaves (Fig. 6g). The substantial increase in flavonoid content in transgenic lines confirms the validity of the elemental assessment of the improved vacuum-infiltration system. Unfortunately, the transgenic tomatoes were stunted and exhibited a dwarf phenotype (Fig. 6b), which hampered the observation of fruit phenotypes.

### Production of scutellarin based on the improved vacuum-infiltration system

**Figure 7:**
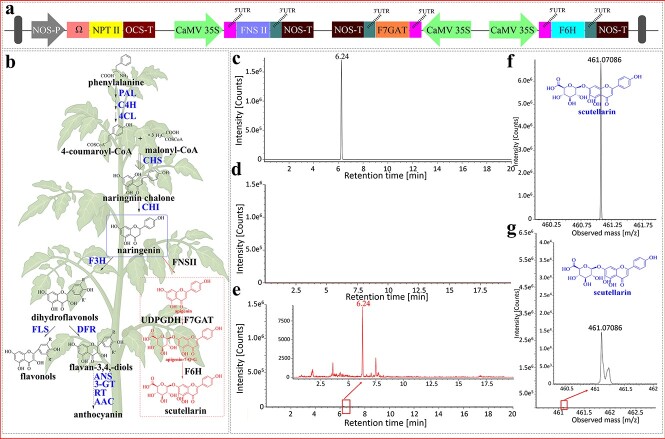
Scutellarin accumulation in vacuum-infiltrated tomato leaves. **a** T-DNA region of the multi-gene vector pAGM4723. **b** The flavonoid biosynthesis pathway (depicted in black) and the introduced scutellarin biosynthesis pathway (depicted in red) in tomatoes. **c**–**g** UPLC-MS analysis of scutellarin in WT and vacuum-infiltrated tomato leaves. The EIC (m/z = 461.0708) of the scutellarin in the standard sample (**c**), WT (**d**), and vacuum-infiltrated tomato leaves (**e**). MS fragmentation patterns of (−)-scutellarin standard (**f**) and (−)-scutellarin in vacuum-infiltrated tomato leaves (**g**).

In order to produce scutellarin in tomatoes, the secondary metabolic pathways of *S. lycopersicum* and *Erigeron breviscapus* were analysed (Fig. 7b). The deficiency of *F6H* and *F7GAT* in *S. lycopersicum* resulted in the inability to produce scutellarin. Therefore, the introduction of *F6H* and *F7GAT* structural genes in *S. lycopersicum* is necessary. In *S. lycopersicum*, the downstream metabolic pathway using naringenin as a substrate is divided into two main branches. One is the formation of flavonols and anthocyanins catalyzed by F3H; the other is the formation of apigenin catalyzed by FNSII. Thus, increasing the expression of *FNSII* could enhance the metabolic flux of baicalein biosynthesis. Therefore, we constructed a pAGM4723 vector containing the quadruple expression cassette (*NPTII* is a resistance gene, Fig. 7a). The methanolic extracts of the leaves of vacuum-infiltrated as well as WT plants were analysed for scutellarin quantification through UPLC-MS. As expected, the extracted ion chromatogram (EIC, m/z = 461.0708) of vacuum-infiltrated leaves showed a distinct peak at 6.24 min (Fig. 7e), which was in the same position as the only characteristic peak of the authentic scutellarin sample (Fig. 7c); however, the presence of this characteristic peak was not detected in the EIC of the WT leaves (Fig. 7d). These results suggested that high-value scutellarin was produced in tomato leaves. This conclusion was also confirmed by mass spectrometry, in which the fragmentation patterns were consistent with the scutellarin standard (Fig. 7f and g).

## Discussion

Given that tomato is a crop model with fleshy fruits and has extensive germplasm resources, complete genome, transcriptome, proteome data, and well-established genetic transformation methods [36], currently, a series of achievements have been achieved in both fundamental and applied research of tomato. For instance, tomatoes are often used for the production of various plant natural products (PNPs) [37]. Notable cases include the deletion of C-terminal autoinhibitory domain of glutamate decarboxylase (SlGAD) which resulted in a 7- to 15-fold increase in γ-aminobutyric acid (GABA) content in tomatoes [38]. Additionally, using CRISPR/Cas9-mediated gene editing technology, tomatoes were enriched with 7-dehydrocholesterol (7-DHC), which is able to be converted to vitamin D_3_ when exposed to sunlight [2]. However, most of these studies require stable transformation of specific genes in tomato, the process of getting transgenic plants remains laborious, time-consuming, and costly [26]. Therefore, rapid, efficient, and alternative methods are warranted for the tomato biological study. The emergence of *Agrobacterium*-mediated transient expression system provides a novel strategy to address this problem. Unfortunately, early studies focused primarily on syringe infiltration of this plant, which does not completely escape the structural limitations of the tissue and does not produce the desired results [25–27]. Although there are also a few reports on the establishment of vacuum infiltration in tomato, they are only applicable to young leaves or detached tissues [28, 29, 31]. At present, the limited applicability and the tendency of detached tomato tissues to rot after *Agrobacterium* infiltration are the major challenges for the tomato vacuum-infiltration system [30].

In our attempt, we established an efficient vacuum-infiltration system based on a self-developed vacuum transformation device (Fig. 1b). The improved vacuum-infiltration system achieved a number of technological advances over the existing vacuum infiltration methods developed in tomatoes, including the following. Firstly, our self-developed vacuum transformation device can generate stronger suction force, which could create micro-wounds in the plant tissue when bubbles are released. Such phenomena may allow *Agrobacterium* to better access and infect the plant cells; in addition, the wounded tissues may secrete numerous phenolic compounds, which act as inducers of the T-DNA transfer process and can significantly improve the transformation efficiency of *Agrobacterium* [39]. Secondly, the improved vacuum-infiltration system could achieve vacuum infiltration of all attached tissues of Micro-Tom because the vacuum chamber could provide the space required for all growth stages of Micro-Tom (Fig. S3, see online supplementary material). This is also the first report of vacuum infiltration of attached tomato fruits. Thirdly, it was demonstrated that the improved vacuum-infiltration system is not only suitable for model tomato but can also be used in commercial tomato (Fig. S2, see online supplementary material). As a nonionic surfactant, Silwet L-77 has been shown to improve cuticle penetration on plant surfaces [6]. Finally, to overcome the limitation of infiltration efficiency by the cuticle of tomato leaves, Silwet L-77 was also successfully used in the modified vacuum-infiltration system and significantly increased the transformation efficiency of *Agrobacterium* in tomato leaves (Fig. 2c).

To evaluate the feasibility of the improved vacuum-infiltration system in tomatoes, stability, reliability, and repeatability were evaluated by a series of experiments, including those involving fluorescent protein, GUS, and *RUBY* reporters (Figs 2 and 3). Comparing our results to those of previous transient expression studies in tomato [16, 17], the improved vacuum-infiltration system enabled global expression of the gene in the leaves (Fig. 2b and d). Although fluorescent proteins and GUS are two commonly used reporters for monitoring gene expression in plants, their application in tomato fruit is not convenient, probably due to their structural properties. The *RUBY* reporter is a new tool for visualizing gene expression without chemical treatment or special equipment that has been used successfully in several plant species [33]. As shown in Fig. 3c, the leaves of *N. benthamiana* showed an intense maroon color, which is consistent with the phenotype in previous studies [20]. By contrast, although our results demonstrated the applicability of the improved vacuum-infiltration system to tomato fruits, betalain was only detected in the fruit placenta (Fig. 3c), which could be related to the fact that the selected promoter was unable to drive gene expression in the intact fruit. Therefore, regulatory elements are also one of the important factors affecting the results of transient gene expression.

In addition to refining the protocol for optimal stability and reproducibility, our attention extended to the study of trichomes. Previous studies have demonstrated that some high-value PNPs are synthesized only in plant trichomes [40]. The trichomes of tobacco have been explored as platforms to produce terpenoids by using a trichome-specific promoter [41]. However, relatively few cases of trichome metabolic engineering have been reported in tomato, which is likely due to the lack of efficient and specific regulatory elements. As such, the mining of trichome-specific promoters will facilitate the production of specialized metabolites with high industrial or pharmacological value [42]. In this study, we found that *AaLTPpro* could drive the expression of exogenous genes in the trichomes of both tomato and *N*. *benthamiana* (Fig. 4b), which can be employed as a candidate element to drive the directed synthesis of PNPs in tomato. For example, artemisinin and cannabinoids are two valuable medicinal ingredients derived from trichomes that have been used to treat malaria and cancer, respectively [43, 44]; however, due to the low level of endogenous content, policy control (restricted cultivation), lack of effective heterologous supply, etc., these two metabolites still cannot meet the market demand [44, 45]. Based on our results, it is promising to synthesize artemisinin and cannabinoids in tomato trichomes by expressing key genes in the metabolic pathway under the control of *AaLTPpro* (Fig. 4c), which would also provide an alternative source of these valuable compounds. More importantly, the improved vacuum-infiltration system, as an efficient tool, can also be applied to expand the pool of synthetic biology elements adapted to the tomato chassis, a strategy that can also be replicated in other plant species.

Moving beyond genetic engineering, the significance of modifying plant traits becomes evident when considering flavonoids, an important class of hydrophilic dietary antioxidants that have a negative correlation between their higher intake and decreased risk of cardiovascular, cancer, and other age-related diseases [46]. However, the antioxidant profile of tomatoes presents an intriguing contrast. While lycopene is the most abundant antioxidant in tomato fruits, its flavonoid contents (including anthocyanins) are considered suboptimal [35]. Therefore, the development of flavonoid-rich tomatoes is necessary from the perspective of prevention and protection against chronic diseases. Recognizing this need, researchers have created several new flavonoid-rich tomato varieties through ectopic expression of either a select number of key biosynthetic genes or regulatory elements [35, 47–49]. Among them, TFs can simultaneously regulate the expression of multiple genes in metabolic pathways, which is a more effective tool to enhance the flavonoid content in tomato [47]. However, no studies on the quantitative comparison of these TFs that promote flavonoid accumulation in tomatoes have been reported. Undoubtedly, conducting the assessment using the plant stable transformation approach would be a time-consuming task. In contrast, the use of the improved vacuum-infiltration system in tomato could significantly shorten the research period. Based on the previous reports [50], 12 TFs from different species were selected and randomized into six groups (Fig. 5a). Based on a previously reported approach for quantifying genetic element efficacy through a transient expression system in *N. benthamiana* [20], a similar approach was adopted for tomato in this research. Among the vacuum-infiltrated tomato leaves, leaf 6^#^ (*AmRosea* + *AmDelila*) had the highest flavonoid content, which was 2.11 times higher than that of WT leaves (Fig. 5d), indicating that the combination of *AmRosea* and *AmDelila* can be considered as a candidate element for the development of high-value tomato varieties. As a proof of concept, *AmRosea* and *AmDelila* were stably transformed into tomato. Consistent with the vacuum-infiltrated results, the amounts of flavonoids accumulated in the leaves of the transgenic plants were significantly higher than those of WT plants (Fig. 6g). Although this result confirmed the accuracy of the improved vacuum-infiltration system for evaluating elements suitable for tomatoes, it should be noted that the transgenic plants were unable to thrive (Fig. 6b), which may have resulted from the use of the constitutive promoter CaMV35S that enabled the accumulation of excessive secondary metabolites in the plants. A similar result was reported by Gwak *et al.,* who showed that heterologous production of the ginsenoside saponin and its precursors in transgenic tobacco impaired vegetative and reproductive growth [51]. In contrast, transgenic tomatoes driven by a fruit-specific promoter (*AmRosea*-*AmDelila*-OE) were able to grow normally and produce fruits with high flavonoid content [48], and several studies have also demonstrated that the use of fruit-specific promoter to control compartmentalization synthesis of target products in tomatoes would avoid the possible toxic effects of excessive products on plant development [52, 53]. Therefore, the mining and use of tissue-specific promoters will be helpful in advancing bioengineering research in tomato.

In recent years, synthetic biology research using plants as chassis has made some progress in producing high-value PNPs [2, 54, 55]. However, limited by the lack of effective standardized elements, plant synthetic biology research is still in its infancy [56]. The mining of tomato trichome-specific promoters and the characterization of TF combinations in this study highlighted the potential of transient expression methods for high-throughput screening of synthetic biology elements. With the help of this efficient method, more novel plant varieties with nutritional or medicinal value will be created in the future.

The main strategies for increasing or producing bioactive compounds through exogenous systems involve reconstruction of biosynthetic pathways, overexpression of crucial TFs, and blocking of competitive pathways. Plant cell factories established on this concept efficiently synthesized a variety of high-value natural compounds and effectively increased the overall yield of PNPs [18, 52, 54, 57]. Scutellarin is a flavonoid extract derived from the traditional Chinese herbal plant *E. breviscapus* and is commonly used to treat of cardiovascular and cerebrovascular diseases [58]. However, extraction from *E. breviscapus* remains the main source of scutellarin, greatly depleting the wild resources of this species. Therefore, the development of alternative routes for the supply of scutellarin is essential. The biosynthesis of scutellarin is well elucidated and only needs three enzymatic reactions to convert naringenin (NAR) into scutellarin (Fig. 7b) [59]. NAR, an important precursor for the synthesis of scutellarin, has been identified in tomatoes [60]. To achieve the biosynthesis of this metabolite in tomatoes, the introduction of the three crucial genes (*EbFNSII*, *EbF6H*, and *EbF7GAT*) is proposed. Although *EbF6H* belongs to the cytochrome P450 family [59], which typically requires the participation of cytochrome P450 reductases (CPRs) to function in the electron transport chain, several studies have suggested that P450 might work well even without the assistance of CPRs [54, 61]. So, in order to reduce the vector loading and increase the efficiency of gene expression, CPRs were not transferred into tomato leaves. A similar strategy was also used in our previous study, which provided theoretical support for the successful synthesis of scutellarin in tomato [17]. In this study, we also demonstrated that scutellarin can be synthesized via the reconstituted metabolic pathway in tomato leaves, thereby providing the first report of the heterologous synthesis of scutellarin in tomato (Fig. 7). The rapid and efficient transient production method presented here may become a promising strategy for the heterologous production of high-value PNPs in plants. In fact, transient expression and stable expression have their own characteristics. Undoubtedly, stable expression is the conventional way to produce PNPs due to the ability to create plants with permanent and sustainable productivity. Given the slow growth or difficult regeneration of some plants, obtaining target PNPs by establishing transgenic plants can be challenging. Therefore, the development of transient expression methods provides an opportunity to address these issues in order to meet the continuing demand for currently used and novel PNPs.

In conclusion, the improved vacuum-infiltration system can be used for element evaluation and screening, which is another powerful tool for plant biotechnology research. Meanwhile, the successful establishment of the improved vacuum-infiltration system in tomatoes not only represents a significant advancement in transient expression methods, but also serves as a reference for the development of similar approaches in perennials and plants that are recalcitrant to regeneration.

## Materials and methods

### Plant material and growth conditions

The seeds of Micro-Tom tomato (*S. lycopersicum* L.) were obtained from Ball Horticultural Company (https://www.ballhort.com/) and sown in soil-filled pots (diameter: 13 cm, height: 18 cm). *N. benthamiana* seeds were stored in our laboratory. Plants were cultivated under standard greenhouse conditions (25 ± 1°C temperature, 16 h light/8 h dark cycle, 109 μmol m^−2^ s^−1^ light intensity, and 60% relative humidity) for all growth stages. The commercial tomato fruits (‘Emerald’ and ‘Provence’) were gifted by Ruohe Yin (Shanghai Jiao Tong University, Shanghai, China).

### Plasmid construction, expression cassette assembly, and plant stable transformation

To construct multiple expression cassettes, Golden Gate cloning (GGC) was used for multi-level assembly. For level-0 components, CaMV35S-P + 5 U and 3 U + NOS-T were PCR-amplified from the pEAQ-HT-DEST plasmid using Taq DNA polymerase (GenScript Biotech Corporation, Nanjing, China) [62]. The genes involved in this study were derived from two different modalities (PCR amplification or synthesis by the Sangon Biotech (Shanghai) Co., Ltd; Table S2, see online supplementary material), and their ORFs were supplemented with specific fusion sites at both ends (Table S1, see online supplementary material). Level-1 (pICH47732/41/51/61/80) and Level-2 (pAGM4723) vectors were kindly provided by Prof. Sylvestre Marillonnet [63]. The detailed assembly process is shown in Fig. ure S1 (see online supplementary material). For the GUS staining assay, the upstream promoter sequence (Table S3, see online supplementary material) involved in this study was amplified and ligated into pCAMBIA1391Z plasmid.

For plant-stable transformation, a binary vector (pAGM4723) containing CaMV35S-P + 5 U::*AmRosea*::3 U + NOS-T cassette and CaMV35S-P + 5 U::*AmDelila*::3 U + NOS-T cassette was constructed using standard Golden Gate assembly techniques (Fig. 6a; Fig. ure S1, see online supplementary material). The final plasmid, pAGM4723-35S-*AmRosea-AmDelila*, was transferred into *Agrobacterium tumefaciens* strain GV3101. The tomato cultivar Micro-Tom was stably transformed using the previously described *A. tumefaciens*-mediated method [64]. Table S1 (see online supplementary material) contains all primers used in this research.

### Preparation of agroinfiltration

GV3101 (pSoup-p19), a strain of *A. tumefaciens*, was used to deliver Ti plasmid into tomato leaves. *A. tumefaciens* was cultured overnight (200 rpm, 28°C) in 10 mL of LB medium supplemented with 40 μM acetosyringone (AS, Beijing Solarbio Science & Technology Co., Ltd, Beijing, China), 10 mM 2-(N-morpholino)ethanesulphonic acid (MES, pH 5.6), and appropriate antibiotics. Overnight cultures were centrifuged at 4000 rpm for 10 min and resuspended in agroinfiltration buffer (200 μM AS, 10 mM MgCl_2_, Beijing Dingguo Changsheng Biotechnology Co., Ltd, Beijing, China) at an OD_600_ of 0.8. The *A. tumefaciens* suspension was kept at room temperature for 3 h before infiltration.

### Efficient agroinfiltration of tomato leaves and fruits

Well-expanded tomato leaves (9-week-old) were selected for agroinfiltration. Prior to agroinfiltration, *A. tumefaciens* suspension was supplemented with Silwet L-77 (1/5000 v/v, Coolaber, China) and transferred into a 50-mL sterile centrifuge tube. As shown in Fig. 1a, the leaves were immersed in the centrifugal tube, which is secured in the clamp of the iron support stand. Subsequently, the vacuum transformation device was used for agroinfiltration, which was designed and manufactured by our team in collaboration with Shanghai Wonbio Biotechnology Co., Ltd (Model: PTS-50, Shanghai, China, Fig. 1b). In this study, plant tissues were kept in the vacuum chamber at 100 mbar for 20 s to ensure that the air inside the tissues was completely expelled. Finally, the release valve was opened (turn counter-clockwise) to release pressure, allowing *Agrobacterium* to penetrate the interior of the plant tissue, and the above operation was repeated twice. Before the corresponding experimental analyses, infiltrated plants were incubated in a dark room for 24 h and then in a greenhouse for 3–5 days (Fig. 1c and d). The abovementioned process is also applicable to fruits, with the exception that the fruits need to be pierced with a needle prior to agroinfiltration to facilitate the infiltration of *A. tumefaciens*.

### Histochemical GUS activity assay

The GUS staining assay was performed by incubating tissues in GUS staining solution (1/1000 v/v Triton X-100, 0.1 mol L^−1^ sodium phosphate buffer, pH 7.0, 2 mM X-Gluc, TargetMol, USA) for 12 h at 37°C in dark conditions. Tissues were then destained with an ethanol solution at 60°C to remove the chlorophyll. Stained tissues were photographed using a Leica microscope (Olympus, Tokyo, Japan) coupled with an insight digital camera.

### Confocal microscopy

The infiltrated leaves were used for fluorescence observation. An upright confocal microscope, the Leica TCS SP5-II (Leica Microsystems Inc., Bufalo Grove, IL, USA), was used to visualize the protein expression intensity. The excitation and emission detection wavelengths for YFP, CFP, EGFP, and mCherry were set as follows: 514 nm–527 nm, 456 nm–480 nm, 484 nm–510 nm, and 587 nm–610 nm, respectively.

### Total flavonoids extraction and content determination

Total flavonoids were extracted according to the method described by Yao *et al.* [17], with some modifications. In brief, 0.02 g of lyophilized powder was sonicated in 1 mL of 100% methanol for 30 min and then centrifuged at 12 000 *g* for 10 min. The supernatant was transferred to a new tube, and the precipitate was resuspended with 1 ml of 100% methanol and centrifuged again (12 000 g, 10 min). The combined supernatants (2 mL) were used for flavonoid chromogenic reactions and content determination assays. For TFC, a plant flavonoids assay kit (A142–1-1, Nanjing Jiancheng Bioengineering Institute, Nanjing, China) was used.

### UPLC-MS analysis of scutellarin

A total of 0.05 g of lyophilized tomato leaves were ground and then extracted twice with 100% methanol. The combined supernatants (2 mL) were filtered by 0.22 μm syringe filter (Sartorius, Göttingen, Germany) and analysed by UPLC-MS (Acquity UPLC I-class/VION IMS QTOF, Waters Corp., Milford, MA, USA). The separation of the target substances was achieved on a BEH C18 column (2.1 × 100 mm, 1.7 μm, Waters), which was kept at 45°C in the column oven. For the gradient elution procedure, 0.1% formic acid in ddH_2_O was used as solvent A, 0.1% formic acid in MeCN as solvent B, and the flow rate was set at 0.4 mL/min. The elution process is as follows: 0–3 min, 5–20% B; 3–10 min, 20–100% B; 10–12 min, 100% B; 12–15 min, 100–95% B; 15–19 min, 95% B. For the detection of scutellarin, 2 μL of sample was injected, and the wavelength was set to 335 nm. The setting of the mass spectrometry parameters and the data analysis were performed according to Xia *et al.* [65].

#### Accession numbers

*AmRosea* (KP311682.1), *AmDelila* (M84913.1), *AtMYB12* (At2g47460), *AtMYB75* (AY519563.1), *SmMYB1* (KF727476.1), *ZmLC* (M26227.1), *ZmC1* (M37153.1), *EbFNS II* (KC521362), *EbF6H* (KU237240), *EbF7GAT* (KU237242), *SlMYB114* (Solyc10g086260), *SlMYB12* (Solyc01g079620.2.1), *SlMYB75* (Solyc10g086250.2.1), *SlAN11* (Solyc03g097340), *SlAN2-Like* (Solyc10g086290).

## Supplementary Material

Web_Material_uhae197

## Data Availability

The datasets presented in this study can be found in online repositories. The names of the repository/repositories and accession number(s) can be found in the article/supplementary data.
