# Supplementary material for: Improvement and application of vacuum-infiltration system in tomato
Source: Hortic Res. 2024 Jul 26;11(9):uhae197. doi: 10.1093/hr/uhae197 (PMC11387009; doi:10.1093/hr/uhae197)
Supplement: Web_Material_uhae197 [file web_material_uhae197.zip › Fig. S3.pdf]

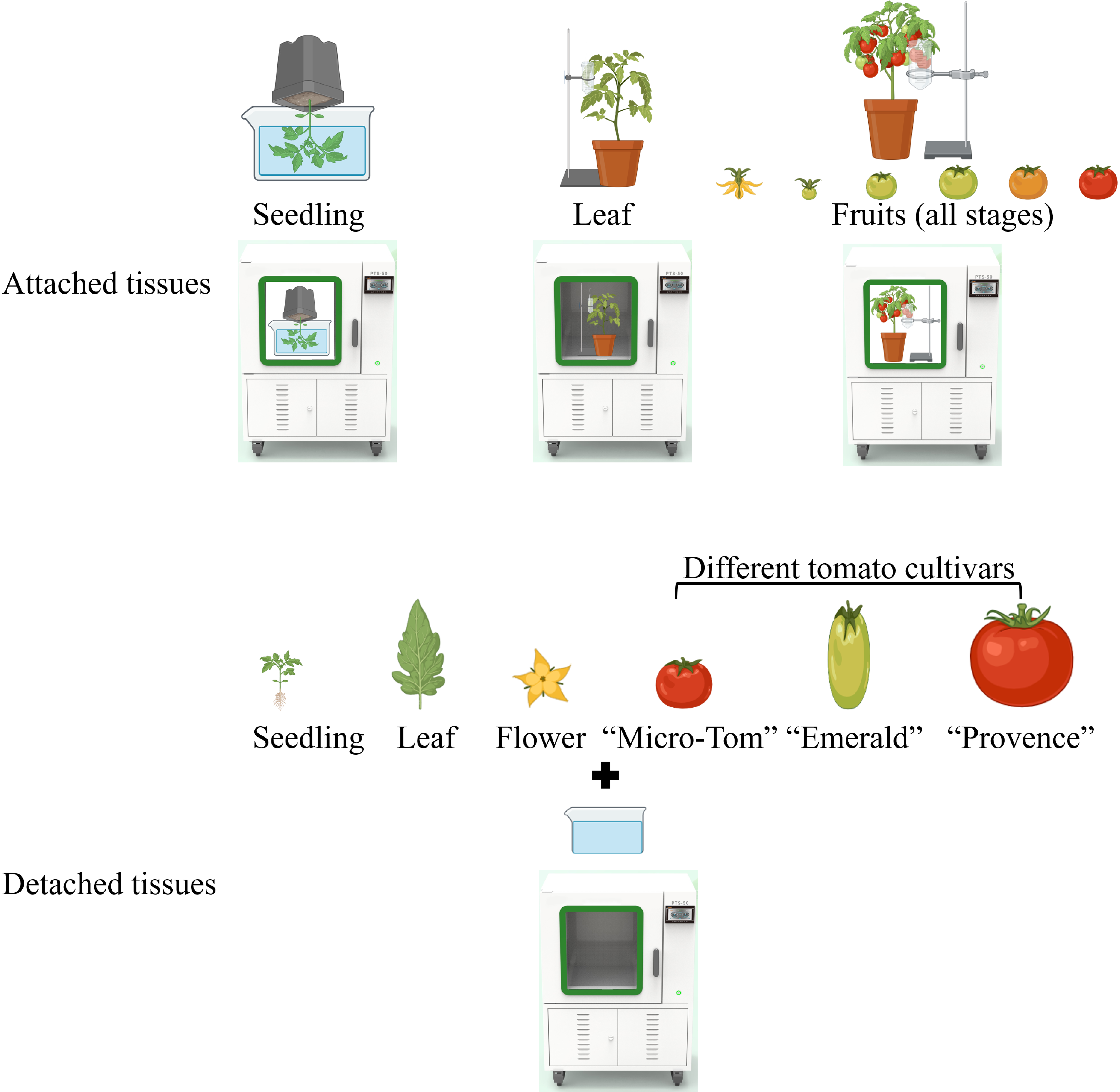
Seedling
Leaf
Fruits (all stages)
Attached tissues
PTS-50
Different tomato cultivars
Seedling
Leaf
Flower
"Micro-Tom"
"Emerald"
"Provence"
Detached tissues
