# Supplementary material for: Improvement and application of vacuum-infiltration system in tomato
Source: Hortic Res. 2024 Jul 26;11(9):uhae197. doi: 10.1093/hr/uhae197 (PMC11387009; doi:10.1093/hr/uhae197)
Supplement: Web_Material_uhae197 [file web_material_uhae197.zip › Table S1. List of primers used in this study.doc]

**Table S1 Primers used in this study.**

| Primer Name | Sequence (5’-3’) | |
| --- | --- | --- |
| EGFP-F | ATCGGTCTCATTGTatggtgagcaagggcgag | |
| EGFP-R | GTGGGTCTCAGCTGtcacgaagccttgtacagct | |
| CFP-F | ATCGGTCTCATTGTatggtgagcaagggcgag | |
| CFP-R | GTGGGTCTCAGCTGttacttgtacagctcgtccatgcc | |
| mCherry-F | ATCGGTCTCATTGTatggtgagcaagggcgag | |
| mCherry-R | GTGGGTCTCAGCTGctacttgtacagctcgtccat | |
| YFP-F | ATCGGTCTCATTGTatggtgagcaagggcgag |  |
| YFP-R | GTGGGTCTCAGCTGttacttgtacagctcgtccatg |  |
| 35S-P-5’UTR-F | GCAGGTCTCAGGAGggaaacctcctcggattcca | |
| 35S-P-5’UTR-R | GAGGGTCTCTACAAatcgaatttgggcagaatatacag | |
| 3’UTR-NOS-T-F | AGCGGTCTCACAGCttaactctggtttcattaaattttc | |
| 3’UTR-NOS-T-R | TCAGGTCTCTAGCGgatctagtaacatagatgacacc | |
| Aa270_pro-_p1391-F | CGGGATCCgacgtacaacactataa | |
| Aa270_pro-_p1391-R | CATGCCATGGgtttggtttgatataagtt | |
| Aa370_pro-_p1391-F | CGGGATCCtattattcgaataaagg | |
| Aa370_pro-_p1391-R | CATGCCATGGgatgatctgtatacaaatg | |
| Aa640_pro-_p1391-F | CGGGATCCgatctttggcattcttggt | |
| Aa640_pro-_p1391-R | CATGCCATGGttctccacttgctattact | |
| Aa820_pro-_p1391-F | CGGGATCCtagtatgaaaatagcag | |
| Aa820_pro-_p1391-R | CATGCCATGGtgattaaacttgttttttc | |
| Aa6601_pro_-p1391-F | CGGGATCCctggaggagagtgtttct | |
| Aa6601_pro_-p1391-R | CATGCCATGGtttaatcaaatcgtttag | |
| Aa6602_pro_-p1391-F | CGGGATCCcaagagagaaaaatgtac | |
| Aa6602_pro_-p1391-R | CATGCCATGGtttaatcaaatcgtttagttag | |
| Aa270_pro_-p0800-F | CGGTATCGATAAGCTTgacgtacaacactataaaaat | |
| Aa270_pro_-p0800-R | ATCCCCCGGGCTGCAGgtttggtttgatataagttg | |
| Aa370_pro_-p0800-F | CGGTATCGATAAGCTTtattattcgaataaagggata | |
| Aa370_pro_-p0800-R | ATCCCCCGGGCTGCAGgatgatctgtatacaaatgc | |
| Aa640_pro_-p0800-F | CGGTATCGATAAGCTTgatctttggcattcttggttag | |
| Aa640_pro_-p0800-R | ATCCCCCGGGCTGCAGttctccacttgctattactt | |
| Aa820_pro_-p0800-F | CGGTATCGATAAGCTTtagtatgaaaatagcagtttta | |
| Aa820_pro_-p0800-R | ATCCCCCGGGCTGCAGtgattaaacttgttttttctt | |
| Aa6601_pro_-p0800-F | CGGTATCGATAAGCTTctggaggagagtgtttctag | |
| Aa6601_pro_-p0800-R | ATCCCCCGGGCTGCAGtttaatcaaatcgtttagtta | |
| Aa6602_pro_-p0800-F | CGGTATCGATAAGCTTcaagagagaaaaatgtacata | |
| Aa6602_pro_-p0800-R | ATCCCCCGGGCTGCAGtttaatcaaatcgtttagtta | |
| 5GT-F | GCAGGTCTCATTGTATGACTGCTATTAAGATGAATACTAAT | |
| 5GT-R | GAGGGTCTCTGCTGTTATTGCAAAGATGGCTCCAACTTA | |
| CYP76AD1-F | GCAGGTCTCATTGTATGGATCATGCTACTTTGGCTA | |
| CYP76AD1-R | GAGGGTCTCTGCTGTTAATACCTTGGAATTGGAATCAA | |
| DODA-F | GCAGGTCTCATTGTATGAAGATGATGAATGGTGAGG | |
| DODA-R | GAGGGTCTCTGCTGTTAAGCAGAAGTAAACTTATAAGAAC | |
| qSlTIP41-F | TCAGTGGGAGGATTGTAAGG | |
| qSlTIP41-R | GGTTCTTTAGACGCCAATGC | |
| qDODA-F | GACATTGGGAGACTGTTAAGCCT | |
| qDODA-R | TTCCTAGCAAGCTCTGGCTCTC | |
| qGUS-F | CTTACGCTGAAGAGATGCTCGAC | |
| qGUS-R | TGATTGATGAAACTGCTGCTGTCG | |
