# Supplementary material for: Improvement and application of vacuum-infiltration system in tomato
Source: Hortic Res. 2024 Jul 26;11(9):uhae197. doi: 10.1093/hr/uhae197 (PMC11387009; doi:10.1093/hr/uhae197)
Supplement: Web_Material_uhae197 [file web_material_uhae197.zip › Table S2. Acquisition of the genes involved in this study.doc]

**Table S2 Acquisition of the genes involved in this study.**

| Gene Name | PCR amplification | synthesis |
| --- | --- | --- |
| *EbFNS II^*^* |  | √ |
| *EbF6H^*^* |  | √ |
| *EbF7GAT^*^* |  | √ |
| *EGFP-F* | √ |  |
| *CFP* | √ |  |
| *CFP* | √ |  |
| *mCherry* | √ |  |
| *35S-P-5’UTR* | √ |  |
| *3’UTR-NOS-T* | √ |  |
| *Aa270_pro_* | √ |  |
| *Aa290_pro_* | √ |  |
| *Aa370_pro_* | √ |  |
| *Aa640_pro_* | √ |  |
| *Aa660_pro_* | √ |  |
| *Aa820_pro_* | √ |  |
| *SlMYB12* | √ |  |
| *AtMYB12* | √ |  |
| *SlMYB75* | √ |  |
| *AtMYB75* | √ |  |
| *SlAN11* | √ |  |
| *SlAN2-Like* | √ |  |
| *ZmLC^*^* |  | √ |
| *ZmC1^*^* |  | √ |
| *SlMYB114* | √ |  |
| *SmMYB1^*^* |  | √ |
| *AmRosea^*^* |  | √ |
| *AmDelila^*^* |  | √ |
| *5GT* | √ |  |
| *CYP76AD1* | √ |  |
| *DODA* | √ |  |
| * The sequence information of both ends of the gene is shown in the following table. | | |
| *EbFNS II* | | |
| ATCGGTCTCATTGTATGAACATGT -- (1541 bp) --TCTACTTAACAGCTGAGACCCAC | | |
| *EbF6H* | | |
| ATCGGTCTCATTGTATGGCATCAA -- (1544 bp) --TACATGTGACAGCTGAGACCCAC | | |
| *EbF7GAT* | | |
| ATCGGTCTCATTGTATGGAAAACA -- (1400 bp) --CGAGTCTAGCAGCTGAGACCCAC | | |
| *ZmLC* | | |
| ATCGGTCTCATTGTATGGCGCTTT -- (1814 bp) --AAGCGGTGACAGCTGAGACCCAC | | |
| *ZmC1* | | |
| ATCGGTCTCATTGTATGGGGAGGA --(803 bp) --CTTGCGTAGCAGCTGAGACCCAC | | |
| *SmMYB1* | | |
| ATCGGTCTCATTGTATGAATAATC -- (752 bp) --CTTGATTAACAGCTGAGACCCAC | | |
| *AmRosea* | | |
| ATCGGTCTCATTGTATGGAAAAGA -- (644 bp) --GGAAATTAACAGCTGAGACCCAC | | |
| *AmDelila* | | |
| ATCGGTCTCATTGTATGGCTACTG -- (1916 bp) --AAGTCTTGACAGCTGAGACCCAC | | |
