# Supplementary material for: Improvement and application of vacuum-infiltration system in tomato
Source: Hortic Res. 2024 Jul 26;11(9):uhae197. doi: 10.1093/hr/uhae197 (PMC11387009; doi:10.1093/hr/uhae197)
Supplement: Web_Material_uhae197 [file web_material_uhae197.zip › Table S3. Promoter sequences of six Artemisia annua genes.doc]

**Table S3** Upstream promoter sequences of six *Artemisia annua* genes.

| *Aa270_pro_* |
| --- |
| GACGTACAACACTATAAAAATTTATATGTGATTCTTTTGTTTATATAGCAAAGTTTATTTACAAAAAATTTAACAAATACCTAACGATTTACACCGCTAGAAACGCGTCCTCATTACAACAAAAAATAAAATAAAATAAAATAAAATAAAAAATAATAAAAAATCTAATATTAATATACTTTTAATATACGTGCGCTTTTTAAAAAAAATTAAAAGAATTAAAATTTAGCATATATTACATATCTAGCAATGTTTTAATAATAAAATATAACCGAACATGCCATAATATTACGAAGAAAGGGAGCCCGAAACTTTAATCGAACAATGACATATCGAGGTTAAAAAGAGTTTGCCAAATTTGGTGAAAGCCCCGCCGCAACGCGGCGAGCTCCTTCTACTTGTATATTTCTATAGCCAAAGATCTTGTCTTGTTTATTCTAGTTTCTCACCTATGATGTTAGCAAATTAAATGAAAAATTAAGTTACTTGGTTTTACATTCTAGATAAAAATAGTACAACATTTACTCAAACAATTTTCACCATATCAAACTTGACATATTCATCAAATGGTGTGTTTAGTTCTTTTAGAAGGTTCGGTTTATGATAAATATAGCAAAGTCAATTTCCATCAAACTCTTTAAAGATATAGGTATAAGGTATGCCATACCAACTATAATCTAGAAATGCCCTTAAAATAAGAACTAAATGGTATTTTAGTAATTACTACAAGAAACTAAGACCTTTTATGTATATGCACAATTATATAAGTAGCAAAACCTTCCCATAATCCTTTCATCCCACTCCAAAAGAACAAAGTAAGAGTACACATTTGCAACTTATATCAAACCAAACATG |
| *Aa370_pro_* |
| TATTATTCGAATAAAGGGATACCAATATGTGCTTGAATGAATCTTTTAATAAATAACCGGTGACACTTGTTTATAATTGCGCATATTTGAGTACCCAAGTGTTTTTTTTTGGTGTTGACATTTTATGCCCGAGTATATATATATTAAGTTCGGAATTGCGTTGACCCTTGCGCTATTTGAAGTCTAGACCATATATTGTAAGCCTGGCAATGTTTGTTTGCGTATTTACCTTATGGTTTCCGAGCTTGCTCCCGGTAATGCGTTTCTGAGTTCATGTATATGATGTTGATGTTCTAGATTCTACACCCTTTACTTGTTTTACCTTAACCATGTTTCTTTGACTGTAAAGAAAAGACGTACTGTATGTATATCTGACCTTTTGAGAGAAGGGCTATTCATCTTTAGTCCTGTCTTAGCTGAATACTCGCAATACTACTGTTGATACTAAACCAGGAAATATAAGCTTTAGCGCATTGTATTACGCTATATTAGTAGCGAGGTCACATATTTGATTATAAGTTCAAACATAGGCTTGTTATCTGAAAGTATGTATCTGTGACATAATTTGCGAGGTTACTTGGTTTTTAATATCAGTACTGAAGATGATTGCTTTACCAACAATAAAAATCTGATTGCATCATTTTATTTGGAGATGTATCCTGTTTAATCGTGTCTTAAAGTTCATCGTTAATGTATTCTTTGCATTTGTATACAGATCATCATG |
| *Aa640_pro_* |
| GATCTTTGGCATTCTTGGTTAGTTGGCACTAACAATAAATAAACAAACAAATAAACTTGCTATTTTTTATTTATTTAGTTGTAAGATGTGCTTAGTTCGTCATCATGGGAGAAAAAGGGCATGCCCAACCATAATCCCATATTACCGCATCTAATCTGCCAACTTTTTTTTCTTCAGACAAAGGTCACACATAACAAATATCATTTTATTATTAAAAAAAACAAAACAAAAGAAAAGAAAACTATGACATAAAATAACTAAATAAATAAAAAAGAATATGCATGATTTGATATCAGTTTTAAATTATTTGATGTTTATTTTAAATATAGTTAAAATATCGCTTGGTATATCTTTATATCATATTAGTTAAGTTTTCCCATGTCTTTAAGAATTAACAAGAATATTTATGAACAATATGTTATTTTTTATAAAAGAGCAATTTGGTTAAATATATAATATTACAAATCTACAACCTTGCTAGTGATTTATGTAGGAGCAAAAAAGAAATTCCAAAATTGTGAGCTACAATTTTAAATAATATTAAATTATTCTAATTAATGTTTAGTTGATTACAATAGACTTTTGTGCATGTATCAACTCTATATAAACCAACTAATAAATCCCCAATAGTTACCAAAACATCTCTTAATATTTCCTCTACCCACCAAGCAGAGAGAATTAAAGTAATAGCAAGTGGAGAAATG |
| *Aa820_pro_* |
| TAGTATGAAAATAGCAGTTTTACTTTTAACTAATTAATTATTGTTTACTAAAAATTGCAATAACAATTTCAAGTAAAAGTAATATAGTATCTCGACGGAATTTCTCTATTCATTTTTAGTGATGAACAATTAACTTGTGACAACCCAAACTAGAAATGTGAATAATAATATTTGGACTGGAGAGTAATTAGAGGTTACAGTCTAAGAACTTAAGTAATTAGAGTAAATTGAAGTTTTTAAAAAGGGAGTTTAAAAACAAAAAACGATAATTTATTTTGAGGTCACAAGTACAAGGATGGATATCTATTTTAGAAGCGAGAAAGCAAAAGTGAGAAATATTTATCAACCGATCGAAAAATATTTGTCAAAACAATATAGATGAAATGAAACAATTTTGATTAATAGACCATATGAGCAGACCTACACGTCTAAGTTAGAGTGTGCACATAAATAGTTTTAAATAGTTAGTGCAATAATTTTAGATTTATATATTAGAGTGCATAATAAAACTAATGAAACGAAATGCATATGCAAATGCAATAAAGTGAGGCGCACAATTTGACTTTGTATTCAATTATCTCATTCCATAATCGATAAAATTTAAAGCCCCTGGACATTGACATCACTATATAAGATGATACACATCTTAACATTTTCATACATCGAAACAAGATACAATACAAAAAGAAAGAAAGAAAAAACAAGTTTAATCAATG |
| *Aa660_pro_* |
| CTGGAGGAGAGTGTTTCTAGTCATATGAAGCCATTCGTAGAGATGGAAATGTAGTATGGCTAGAAGAGCCCTTCGGTGGAGGGGTACGGGTTACTAGTGATGACATTAACCTTATTTGCTATACTTACTGTGGATGCTGGGTGAGTGAGTATGGCAAAAAGGTAAGATGGAAATGTAGCTAGATGGTAGGTGCTATTGGAAGTGAATTGAGGTGTTATTGTAATGTTATGCAATGCTCTATTATTGCTCTGTTTTTGGCCGAGGAAAGTTGAGACAGATATAGTATATAGTATCAACTGATATACGATTAGAGTTAGAATTCAGTTTGAATGTAGTTTCCTATGCTATATAAGGTAAGGGGTATGCCCCAATCCCTTTGGTGATAAACCTTATGATGTAAAGTTGTTGGTTGATTAATATAATTCACATTTGCAAAAAAAATAAAAAAATAAACGATTTCTAATAATTTCAGATTTATTACCCTAACAAAAGTCTACAATTGCTTGCTACATTTGTTAGTCACTTTAAACGAGCTAGCTCATTAATGAATACTAGTTTATTTATTTTATTGTTAAATGCATTTATAATTGTCATCAAGCCTAATAATTCATTAGTTCATTGTGATTAACGTAAAACCATGTGTGCTATTGCATGCTATAAATTGATAAGCAAAGTAACTTGAATGTCATATTTCTTCTAACTAAACGATTTGATTAAAAATG |
| *Aa290_pro_* |
| CGCGGATCCTATTTTGATATATTTTTATAAATTTTTAGTTTTTGCATTTTTAATAGGTTATTTTGATTCAAATTTTTACAACTTTAACCTTCATTTAAATCAATACAAACTTGATTCGTCAAAATTATAACTTTTGAGGGAGTGTTTGACAGGTAAAAAATTTGTGAGGGTCTTTATGCGAGAATAATAATGTCACGGAAAAGTTACGAGAAACCCAAACAAACACATACAACGAACACATATACTTTCGGAGTGTTTTGTAACGAATTCACTAAGCGGGTACGGAATTATGGGAGAGTTGGATTAGCAATTCATTCATTATATTTCTATTATAACTATATTTTTTTTCGCCGCCGCTGTGCGCGGGTATCCAACTAGTTTGAAATATGTGGCTACAATTTCTTCTCACTCTTATCAATTCCATAATTCTTCTCACTAGATCCATACTCTTACATTGTTTACAAACATTCCAACCCAACAACTCTATATATGAACAACGGATTAGCAAATTTTCTTTCAGAAATAACATTGCATTTTTTGGCATAGTAGCTATAAAACTCATTATTAGGCGACATAAAGATTACAAAATTACGTTTGTTTGATCTGTTTAGGGGTGGGAATTAAATAACTTGCGATTATGCTTATATTGATGCGATTATAGTAGCAAAAATCTTTCACAAAGTCAACTTGCATAGCTCAGATTTTCAATGATTTTCAAAACACTCTCTAGGCTTTGCTATTGAATGGAAATGTATAAGCTTTATTGATTTGAGTACCTAGCCAGCTAGTGTATTCATAACAAAAAGAATTTTGGAATTTGAAGATCATTATAACTTGCGATTATGCTTATATTGATGCGATAATGCTTATATTCTGAGATTTTGGGGCCTTGTGAGCATATGCGATCCTTACTGTTTTGTTAAAGAAAAACTTATACACGGTGATGGGTTTCTTTTAGCCATAGCAAACAAATAGCCAGTGTACACGGTTAAACGAATTAGTTATATCTCTCTTTAGGTTACCCGGAAGGGAAAGTTTATGATTCATATGTACCCGGCCAGTCTAACCGCGGTATTTCCGTGACTATTTCCGAACCCTTCCCGGCCACCCTATAGTTTTTTAGTGAGGTTTAAACCGGCCTGATACTTCTTGTAAGGACCTTAAGATGTCAGACCATTAGACCACCTTCATTGGTGGTTAACCAAGAGCCGGTGTGTTTTGACTAATATATTATGCCCCTTCAGATCGAGCTGAAATAATTTGAATCGTGGGACAATGTGTACAATCTCAAAGGGTCGCTACAGTTGCTCTTTGTGGACTTTAATGAAGTTCGGTCTGTCAATTTAATAGATCATCAGCATATTCTGCTTCTAACGCTTCTATATTAAATAGATCAATATTCAATACCGAACTGCATGACTTATATACCGGTGCAGTGGGAGGAACTTTACATTGACGAGAATTGATCGAGAAGGCTAAAAATTAACTGCTATAGGTTGATATATTAGAAATTTTGTTTGGACGTACATGTGAAAGATATAGGCCAAAGATAATATAGGTCGAAGAAAATCTATGTGCATGCATGTAAATGACTGCTGGGCTCTTAATTTGTATTAATGATCTGAAATTAATTTAAATTTCCTCCTTTGTGATCCTTCTATAAATATACAAGTTTACCACACTGATTTCTTCATCCTTCAACTAAATATTTTCTGATATAAAAAGAAATTAAGATCGCCATGGCATG |
