# Supplementary figures and images for: Improvement and application of vacuum-infiltration system in tomato
Source: Hortic Res. 2024 Jul 26;11(9):uhae197. doi: 10.1093/hr/uhae197 (PMC11387009; doi:10.1093/hr/uhae197)

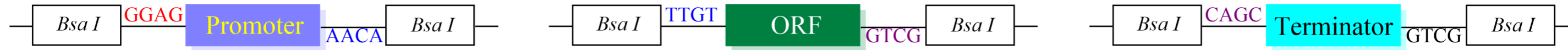

Level 0

PCR Products or Synthesis

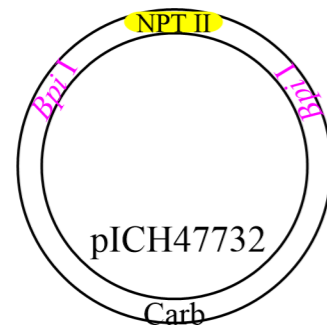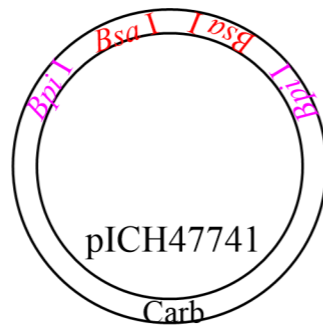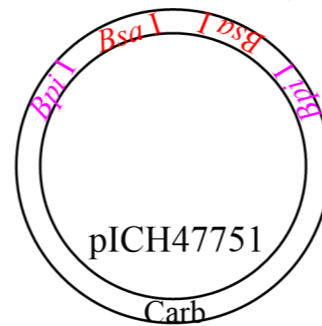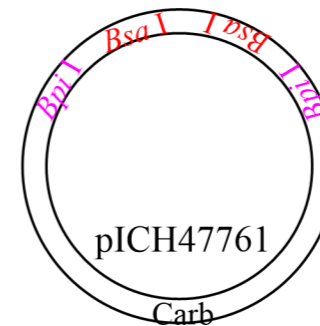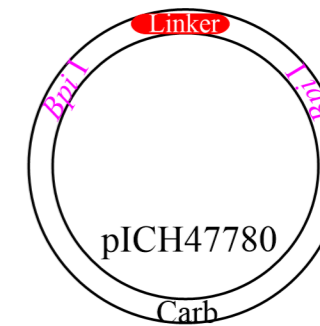

Level 1

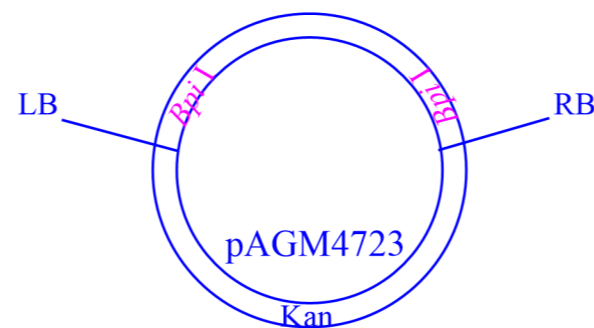

Level 2

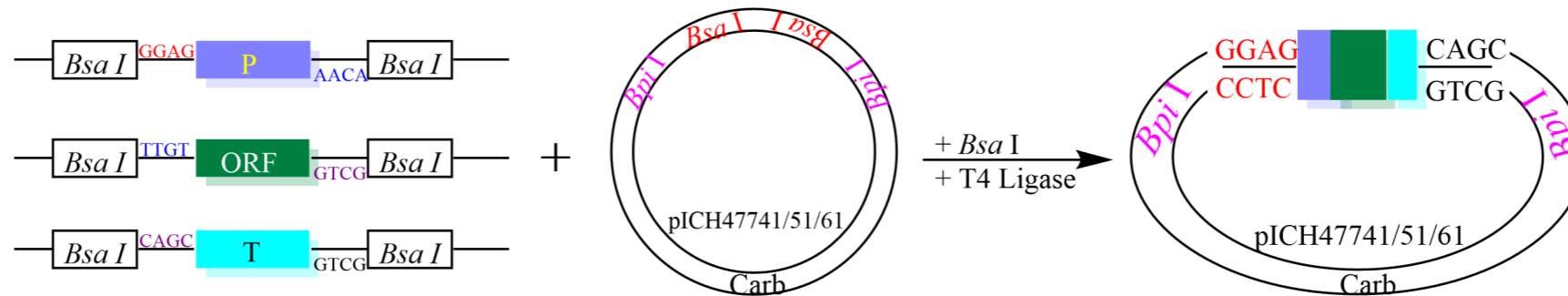

Level 1

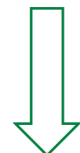

Level 2

Level 2

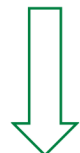

Level 3

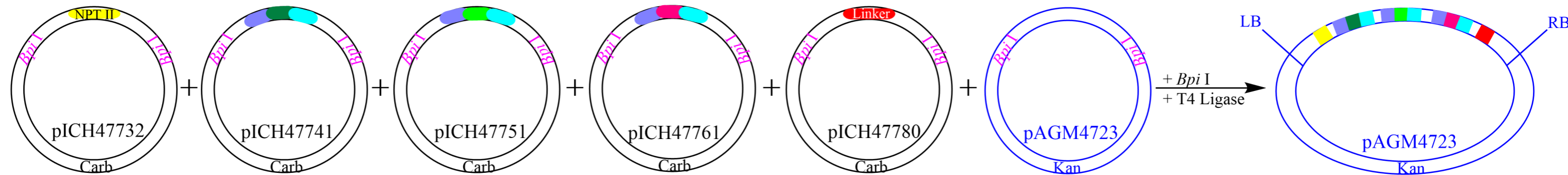

Supplement: Web_Material_uhae197 [file web_material_uhae197.zip › Fig. S1.pdf]

Control

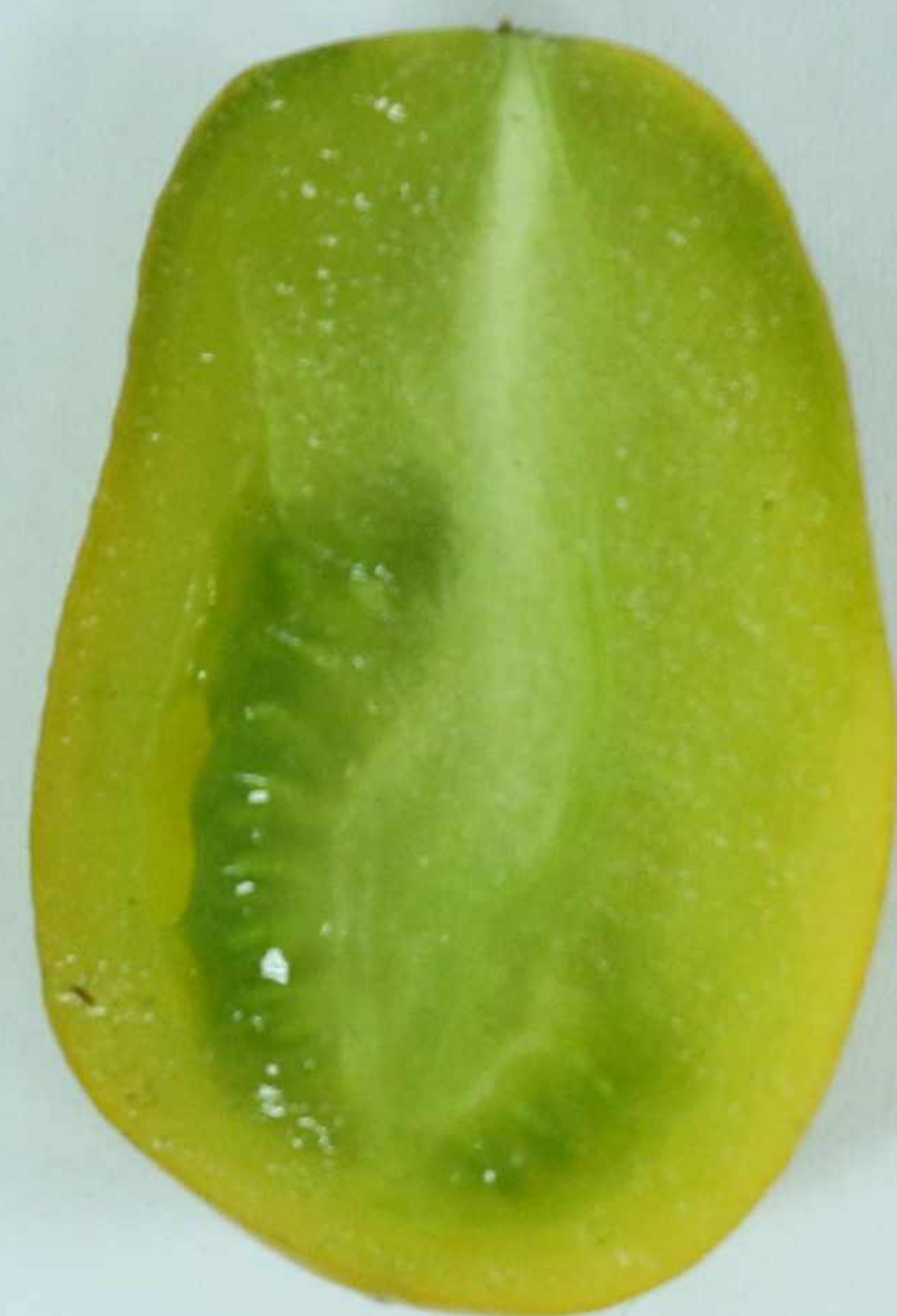

Infiltrated

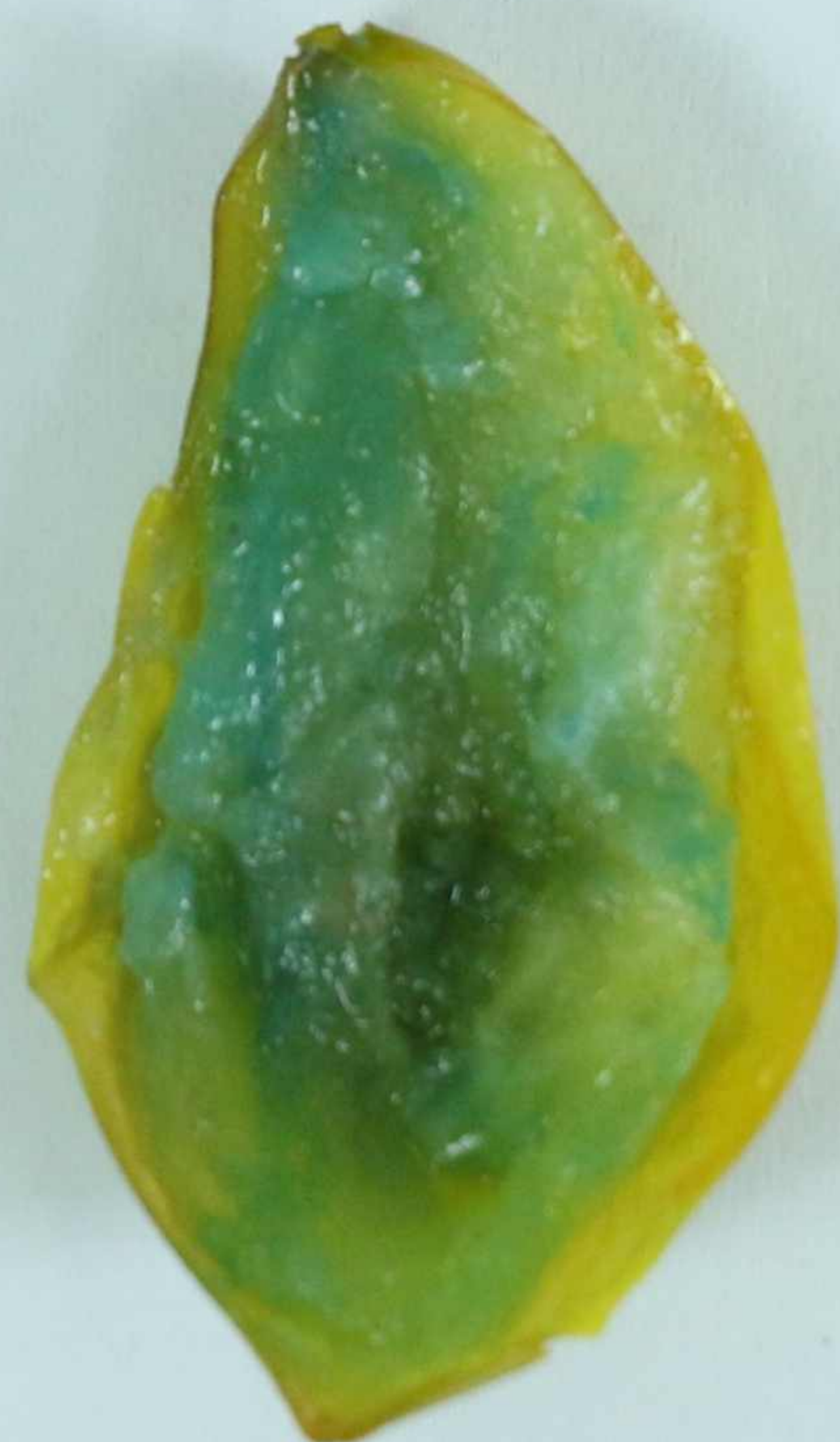

“Emerald”

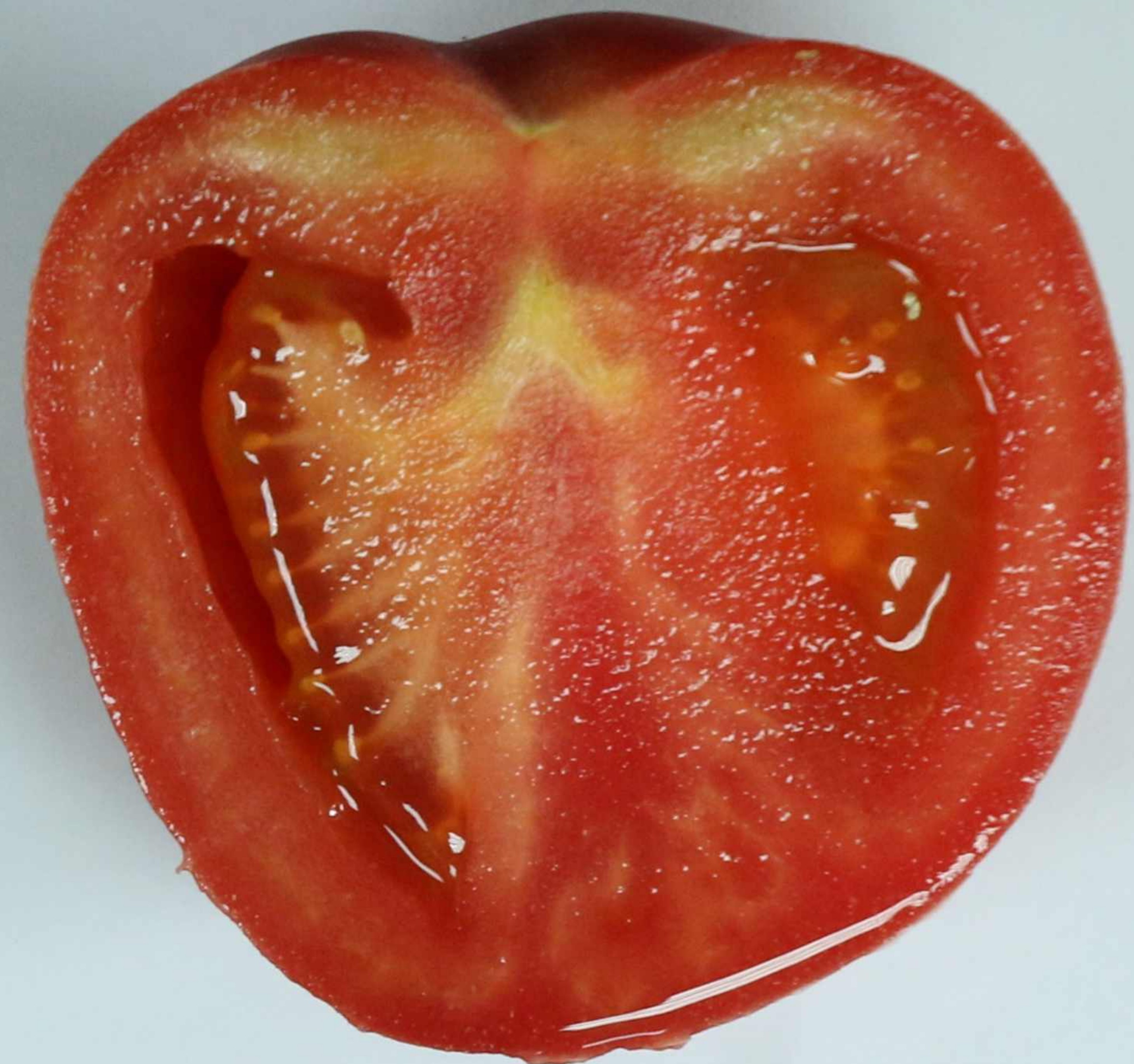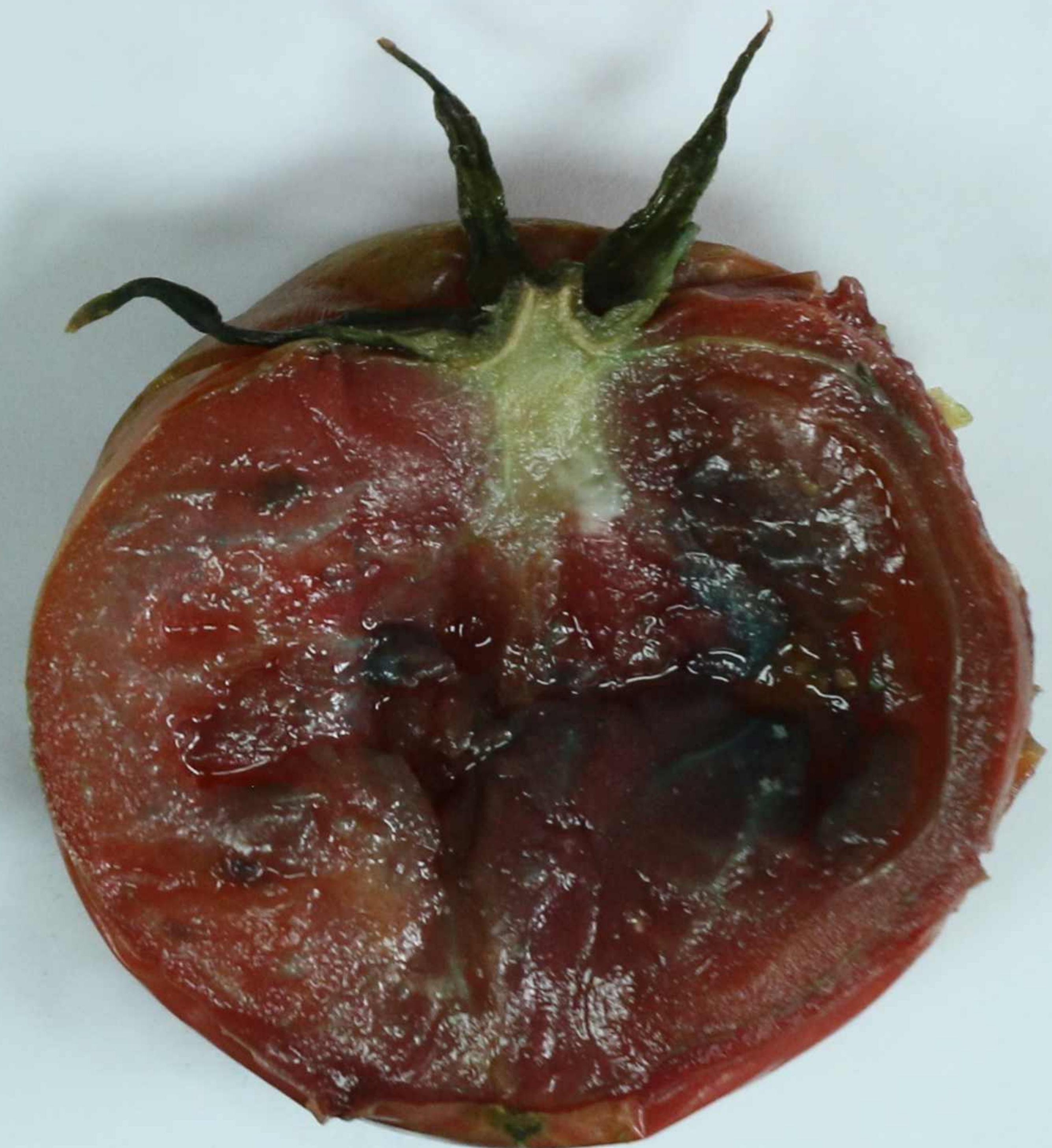

“Provence”

Supplement: Web_Material_uhae197 [file web_material_uhae197.zip › Fig. S2.pdf]
